# Mg,Si—Co-Substituted Hydroxyapatite/Alginate Composite Beads Loaded with Raloxifene for Potential Use in Bone Tissue Regeneration

**DOI:** 10.3390/ijms22062933

**Published:** 2021-03-13

**Authors:** Katarzyna Szurkowska, Paulina Kazimierczak, Joanna Kolmas

**Affiliations:** 1 Department of Analytical Chemistry, Chair of Analytical Chemistry and Biomaterials, Faculty of Pharmacy, Medical University of Warsaw, 02-097 Warsaw, Poland; katarzyna.szurkowska@wum.edu.pl; 2 Chair and Department of Biochemistry and Biotechnology, Faculty of Pharmacy, Medical University of Lublin, 20-093 Lublin, Poland; paulina.kazimierczak@umlub.pl

**Keywords:** nanocrystalline hydroxyapatite, composite biomaterials, raloxifene, drug delivery system, magnesium ions, silicate ions

## Abstract

Osteoporosis is a worldwide chronic disease characterized by increasing bone fragility and fracture likelihood. In the treatment of bone defects, materials based on calcium phosphates (CaPs) are used due to their high resemblance to bone mineral, their non-toxicity, and their affinity to ionic modifications and increasing osteogenic properties. Moreover, CaPs, especially hydroxyapatite (HA), can be successfully used as a vehicle for local drug delivery. Therefore, the aim of this work was to fabricate hydroxyapatite-based composite beads for potential use as local carriers for raloxifene. HA powder, modified with magnesium and silicon ions (Mg,Si-HA) (both of which play beneficial roles in bone formation), was used to prepare composite beads. As an organic matrix, sodium alginate with chondroitin sulphate and/or keratin was applied. Cross-linking of beads containing raloxifene hydrochloride (RAL) was carried out with Mg ions in order to additionally increase the concentration of this element on the material surface. The morphology and porosity of three different types of beads obtained in this work were characterized by scanning electron microscopy (SEM) and mercury intrusion porosimetry, respectively. The Mg and Si released from the Mg,Si-HA powder and from the beads were measured by inductively coupled plasma optical emission spectrometry (ICP-OES). In vitro RAL release profiles were investigated for 12 weeks and studied using UV/Vis spectroscopy. The beads were also subjected to in vitro biological tests on osteoblast and osteosarcoma cell lines. All the obtained beads revealed a spherical shape with a rough, porous surface. The beads based on chondroitin sulphate and keratin (CS/KER-RAL) with the lowest porosity resulted in the highest resistance to crushing. Results revealed that these beads possessed the most sustained drug release and no burst release effect. Based on the results, it was possible to select the optimal bead composition, consisting of a mixture of chondroitin sulphate and keratin.

## 1. Introduction

In many bone diseases involving the formation of cavities and difficulties with regeneration (such as osteoporosis or bone metastasis), bone substitutes are needed to provide structural support for cells and the newly formed osseous tissue, and to induce natural processes of tissue regeneration and development [1,2,3]. Simultaneously, appropriate pharmacotherapy is required to induce bone formation, inhibit osteoclast activity, relieve pain, or provide antibacterial prophylaxis [4,5].

Recently, preparation, characterization, and application of innovative multifunctional biomaterials, with the potential to deliver drugs into the bone tissue, have attracted much attention [6,7,8]. This is thanks to their unique properties such as the possibility for controlled release of drugs, thereby reducing the therapeutic dose and minimizing toxicity [9].

Synthetic hydroxyapatite (HA) with general formula Ca_10_(PO_4_)_6_(OH)_2_ is similar to bone apatite and has high biocompatibility [10,11,12]. These characteristics mean that HA has received a great deal of attention as an inorganic biomaterial in bone tissue replacement. Furthermore, thanks to a high affinity for ionic substitution, HA can be modified in order to obtain additional biological, physicochemical or mechanical properties [13,14]. Finally, due to its high loading capacity, HA could be used for local drug delivery in the treatment and prophylaxis of bone tissue disorders, primarily osteoporosis, bone tumors, and infections [15,16,17].

In the present work, HA modified with magnesium and orthosilicate ions (Mg,Si-HA) was used in order to prepare three-dimensional composite beads for potential use as a drug delivery system into the bone. Mg^2+^ and SiO_4_^4−^ play important roles in the bone mineralization process influencing osteoblast and osteoclast activities, and in bone formation, stimulating collagen type I synthesis [18,19,20,21,22,23].

Raloxifene hydrochloride (RAL) was selected as model drug for the release studies. RAL is a second-generation selective estrogen receptor modulator (SERM) that is used to prevent and treat osteoporosis in postmenopausal women. It exhibits estrogenic effects on the skeletal system and antiestrogenic effects on the breast and endometrium [24,25,26,27]. It thereby prevents the loss of bone mass associated with osteoporosis and decreases the risk of osteoporotic fractures in elderly women. It is interesting that the drug is also used for an adjunctive treatment for schizophrenia or prevention of breast cancer, whilst the latest research reports its potential activity in reducing Covid-19 related mortality [28,29,30].

It is important to note that the current oral therapy with RAL is insufficient because it has poor oral bioavailability (2%) due to hepatic first-pass metabolism and poor water solubility [31]. This fact makes it attractive for controlled drug delivery, which could overcome the limitations of its application in clinical practice.

RAL is classified as a Class II drug according to the Biopharmaceutics Classification System. This means that it has low solubility and high permeability. Therefore, intensive research on new drug formulation or other administration routes to improve its pharmacokinetic properties and bioavailability is required [32,33,34,35,36].

So far, there have been several studies on the local delivery of RAL by calcium phosphate-based materials and there is scope for further development [37,38]. The presented research is a continuation of previous work on beads based on substituted HA, alginate, and chondroitin sulphate, and it involves the introduction of a drug substance whilst investigating the release profile of the drug and substituted ions [39].

To expand the scope of the research, three bead compositions were compared: (i) chondroitin sulphate (CS), (ii) keratin (KER), and (iii) a mixture of both of them in a 1:1 weight ratio (CS/KER). In vitro drug release profiles from beads containing raloxifene, i.e., CS-RAL, KER-RAL, and CS/KER-RAL, were compared. The resulting beads were subjected to in vitro biological tests on osteoblast hFOB and osteosarcoma Saos-2 cell lines. Biological results were compared to control beads without the addition of the drug, CS-c, KER-c, and CS/KER-c, respectively. Porosity and mechanical strength tests were also carried out. 

## 2. Results and Discussion

### 2.1. Bead Morphology

**Figure 1:**
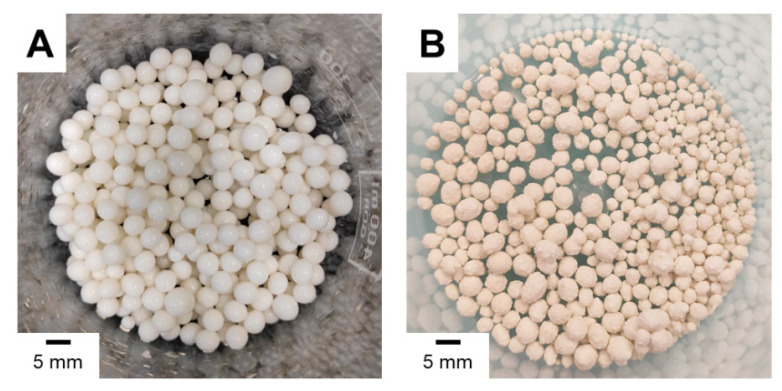
Optical images of representative beads before drying (**A**) and after drying (**B**).

Morphology of representative granules before and after drying is illustrated in Figure 1. As can be clearly seen, the beads show a very smooth surface before drying, whilst dry beads are dramatically rougher. This may be an important advantage to potentially promote the adhesion of bone cells involved in the remodeling of damaged tissue. The obtained granules are of various diameters, from 2 to 5 mm. For further studies, granules with a 3 mm diameter were selected.

**Figure 2:**
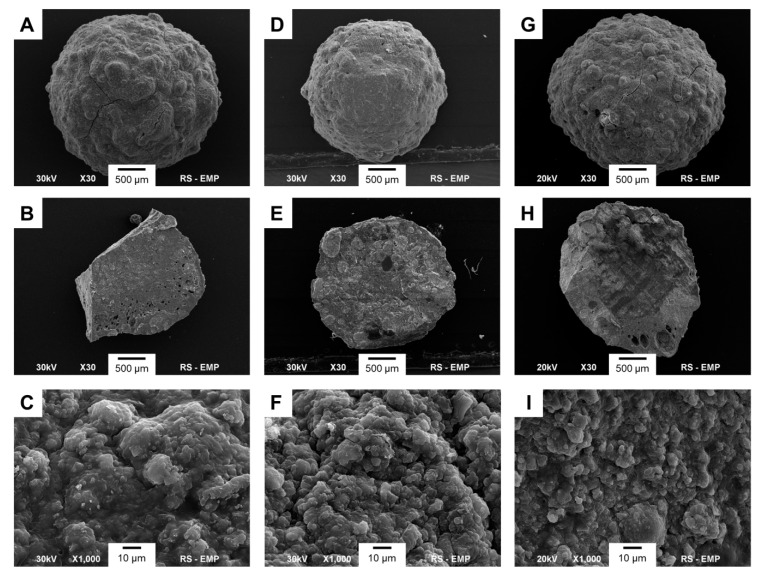
SEM images of CS-RAL (**A**–**C**), KER-RAL (**D**–**F**), and CS/KER-RAL (**G**–**I**). First line: whole bead at ×30 magnification; second line: internal cross-section at ×30 magnification; third line: outer surface at ×1000 magnification.

For morphological analysis, the SEM images taken from three types of beads containing RAL (CS-RAL, KER-RAL and CS/KER-RAL) are shown in Figure 2. All the beads revealed a spherical shape with a rough surface. The CS-RAL beads exhibit the most heterogeneous outer surface, whereas the cross-sectional analysis indicated their relatively dense and smooth interior with numerous fine pores near the outer surface of the spheres. The keratin-containing granules (KER-RAL and CS/KER-RAL) have small indentations on the outer surface, while their graining appears to be more homogenous.

The same measurements were performed for control beads without the drug, but no difference in morphology was observed.

### 2.2. Mechanical Strength and Porosity

The results obtained by the Hg intrusion technique for the samples containing RAL are summarized in Table 1. The apparent and true density values were similar for all the samples (CS-RAL, KER-RAL and CS/KER-RAL) and were in the ranges 1.4–1.5 g/cm^3^ and 1.7–1.9 g/cm^3^, respectively. This indicates that all the samples achieved a high degree of densification during cross-linking.

The obtained beads have a moderate degree of pore surface development (45–71 m^2^/g), while the total pore volume is in the range 0.09–0.15 cm^3^/g, which confirms that relatively compact and dense materials were obtained. The percentage of mesopores between 63 and 74% allows the granules to be classified as mesoporous materials. It should be noted that the KER-RAL sample has the most developed pore surface (71 m^2^/g) and at the same time the highest volume (0.09 cm^3^/g). 

The pore size distribution (data not presented) showed that all the obtained beads mainly contain pores up to 10 nm in size, including micropores. Pores with a larger diameter (over 0.1 mm) were also observed; however, their volume for each sample was only 0.02 cm^3^/g. They may be referred to as inter-grain spaces (cavities), which are probably formed during the cross-linking of the alginate and drying process. 

It should be noted that the degree of porosity and pore size distribution are crucial parameters characterizing drug delivery systems applied to bone replacement and regeneration. The osteogenic properties of the material result from the presence of pores, which facilitate migration of cells, integration with the host tissue, and ensure vascularization. The interconnected pores with a diameter of approx. 100–300 μm ensure cell adhesion and migration, while small pores are required for effective drug delivery systems [40,41].

The average values of the destructive forces for individual samples were also compared. As expected, the beads based on CS/KER-RAL with the lowest porosity (13%) resulted in the highest resistance to crushing (177 N/bead).

### 2.3. Ion Release

**Figure 3:**
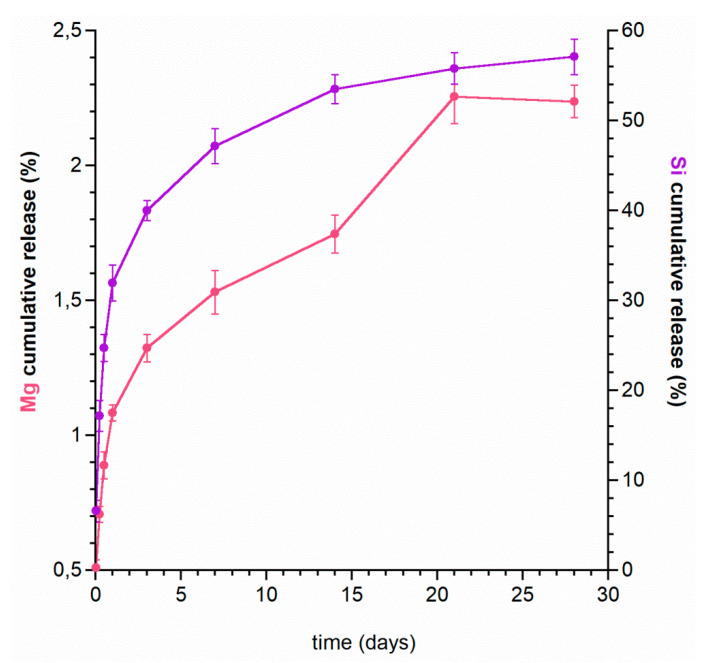
Magnesium and silicon release profiles from Mg,Si-HA powder.

In the first step, the release of magnesium and silicon ions from the starting powder (Mg,Si-HA) for bead fabrication was carried out. Figure 3 shows the cumulative release curves for the ions over four weeks. Interestingly, there are significant differences between the release profile of magnesium and silicon. Magnesium is released gradually and in very small quantities, up to 2.4% of its total content after four weeks. Furthermore, the burst release effect can also be observed for up to 24 h, although, during this time, only slightly more than 1% of the total amount of Mg introduced into the powder is released (see Figure 3). A different release profile applies to silicon, where up to 57% of the introduced element was released within four weeks, and approximately 30% within 24 h. 

In order to explain the ion release results, our earlier studies on the physicochemical properties of the Mg,Si-HA powder should be recalled [39]. The Mg,Si-HA sample was shown to be nanocrystalline with the elongated-shaped crystals with 24 ± 2 nm and 7 ± 1 nm along the *c* and *a* axes, respectively. Moreover, according to ^31^P ssNMR studies, the Mg,Si-HA material, in addition to a well-ordered crystalline core, is characterized by the presence of a non-apatitic layer, called the hydrated surface layer, which is typical for nanocrystalline hydroxyapatite. The ^29^Si ssNMR studies showed that a significant amount of silicon is located just in this surface layer forming “silica gel”. 

A fairly fast release of silicon may be explained by its location on the surface. Initially, the ions, weakly bound with the crystals, pass to the medium, then the ions from the crystal interior are released.

In turn, slow release of magnesium suggests that these ions have been incorporated into the crystalline core and are released along with the slow dissolution of the substituted hydroxyapatite.

**Figure 4:**
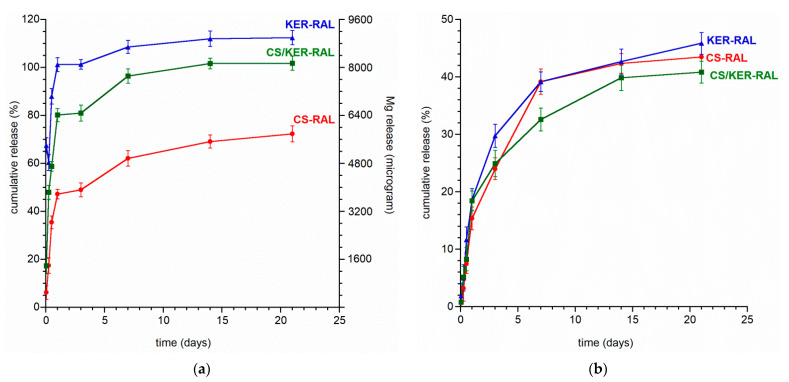
(**a**) The magnesium release profiles from CS-RAL, KER-RAL, and CS/KER-RAL; (**b**) the silicon release profiles from CS-RAL, KER-RAL, and CS/KER-RAL.

Taking into account the small amount of magnesium introduced into the Mg,Si-HA sample (0.26 wt%) (resulting from the substitution limit), we decided to increase its content in the obtained beads by using Mg^2+^ as a cross-linker for sodium alginate. It is worth adding that, conventionally, aqueous calcium solutions are used to cross-link alginates. Figure 4a shows the magnesium release from the obtained beads. As expected, the amount of Mg released from the beads increased significantly compared to that released from the powder. The CS-RAL sample exhibits the lowest amount of Mg released to the medium. It should be noted that this type of bead is also characterized by the lowest porosity. Therefore, Mg^2+^ ions from the cross-linking solution had difficulty accessing the interior of the beads and penetrated them poorly, and, in addition, were released to a lesser extent. During the formation of more porous granules (KER-RAL and CS/KER-RAL), the Mg ions from the cross-linking solution not only binded to alginate but also adsorbed on the bead porous structure. This is why the amount of release magnesium was so high and exceeded 100—100% corresponds to the average amount of cross-linked magnesium ions, however, each type of granule has a different binding capacity.

In conclusion, regardless of the type of beads, it was possible to obtain the initial rapid discharge of Mg^2+^ ions into the medium, followed by stabilization of the release. This is particularly beneficial since Mg ions are responsible for the biocompatibility of the implant materials and bone substitutes.

It is worth paying attention to the release of silicon from the obtained composite granules (see Figure 4b). In the first 24 h, the released silicon was only 15.3–18.6% of the total amount, unlike the release from the Mg,Si-HA powder material, where the value was 31.9%. Moreover, the further release is also slightly slower because the amount of Si released from each type of bead did not exceed 45.9% (vs. 55.9% from the Mg,Si-HA powder). Therefore, thanks to composite production, it was possible to stabilize the release of Si ions and obtain a gradual, extended-release profile. The materials may exhibit a long-lasting osteogenic effect after in vivo implantation which should be analyzed in further research.

### 2.4. Drug Release

**Figure 5:**
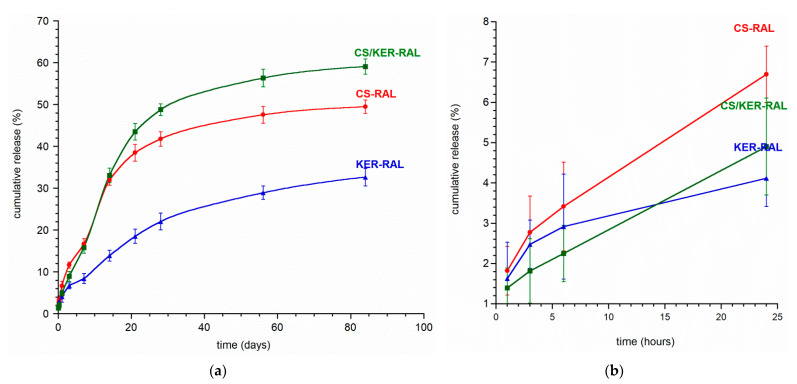
(**a**) Raloxifene hydrochloride release profile from CS-RAL, KER-RAL, and CS/KER-RAL over 12 weeks; (**b**) Raloxifene hydrochloride release profile from CS-RAL, KER-RAL, and CS/KER-RAL during the first 24 h.

After the promising preliminary results of ion release from the powders and granules, an extended three-month release study of raloxifene hydrochloride was performed. The graphs showing the drug substance release profile are presented in Figure 5a,b.

RAL is gradually released from all the obtained granules without a burst release effect, as may be evidenced by the profiles recorded during the first 24 h (see Figure 5b). The release profile of each type of granule is similar, the curves differ mainly in the amount of RAL released to the medium. 

The most favorable release profile was observed with CS/KER-RAL granules containing a mixture of chondroitin sulphate and keratin. The drug was evenly distributed throughout the volume of the granules and was released gradually as the material swelled and slowly dissolved. We managed to avoid disintegration of the granules and the sudden initial burst of the drug, which often occurs in granules containing only sodium alginate [42,43,44]. After 12 weeks, the pellets did not disintegrate, and the amount of released RAL reached up to 60% of the total quantity and still did not plateau. Interestingly, beads containing only keratin exhibit the slowest drug release, while the addition of KER to CS accelerates the release of RAL to the medium.

### 2.5. In Vitro Cytotoxicity Assessment

**Figure 6:**
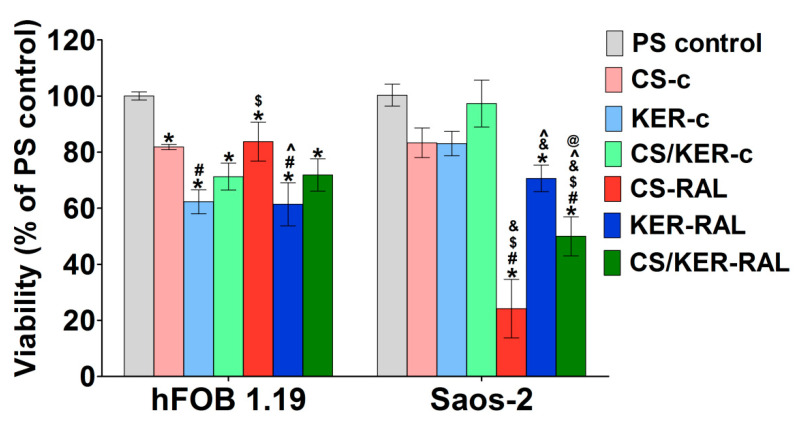
Evaluation of cell viability after 48-h exposure to granule extracts by MTT assay (hFOB 1.19—human normal osteoblasts; Saos-2—human tumor cells derived from osteosarcoma; PS control—polystyrene extract as a negative control of cytotoxicity; * statistically significant results compared to PS control, ^#^ statistically significant results compared to CS; ^$^ statistically significant results compared to KER; ^&^ statistically significant results compared to CS/KER; ^^^ statistically significant results compared to CS-RAL; ^@^ statistically significant results compared to KER-RAL; *p* < 0.05, one-way ANOVA followed by Tukey’s test).

In this study, an MTT assay was performed to assess the cytotoxic effect of 24-h granule extracts on the viability of hFOB 1.19 and Saos-2 cells after a 48-h exposure (see Figure 6). In vitro cytotoxicity evaluation showed that an extract of CS-c and CS/KER-c granules slightly decreased hFOB 1.19 cells viability to 81.83% and 71.27%, respectively. Nevertheless, it should be noted that, according to the ISO 10993-5 procedure, 100% extract of the biomaterial should be considered as non-toxic when the percentage of cell viability is higher than 70%. Importantly, the addition of raloxifene to CS-RAL and CS/KER-RAL granules also exhibited a non-toxic effect on human osteoblasts. The MTT also showed that both an extract of KER-c granules and an extract of KER-RAL granules significantly decreased hFOB 1.19 viability to approximately 60%, suggesting that a reduction in cell viability was not caused by released RAL. It is worth noting that bioceramic-based biomaterials may alter the ionic composition of the culture media via their ion reactivity causing reduction in cell viability [45,46,47]. In turn, CS-c, KER-c, and CS/KER-c granules were non-toxic against Saos-2 cells (cell viability above 83%), whereas extracts of CS-RAL, KER-RAL, and CS/KER-RAL granules caused a significant reduction in Saos-2 viability to approximately 24%, 70%, and 50%, respectively. Thus, in vitro cytotoxicity assessment clearly showed that RAL released from granules was non-toxic against human osteoblasts, but caused a significant decrease in the viability of tumor cells. Considering the results from the cytotoxicity test, both CS-RAL and CS/KER-RAL granules are promising candidates for biomedical applications.

## 3. Materials and Methods

### 3.1. Preparation of Mg,Si-HA

Nanocrystalline, magnesium, and silicon co-substituted hydroxyapatite (Mg,Si-HA) with the nominal composition of Ca_9.87_Mg_0.13_(PO_4_)_5.5_(SiO_4_)_0.5_(OH)_1.5_ was synthesized using the standard precipitation method as previously described [39]. Briefly, Si(CH_3_COO)_4_ and (NH_4_)_2_HPO_4_ aqueous solutions were added dropwise into Ca(NO_3_)_2_ and Mg(NO_3_)_2_ aqueous solution under gentle stirring at pH 10, and the resultant precipitate was left for 24 h for aging. All reagents were purchased from Sigma Aldrich (St. Louis, MO, USA). The resultant powder was subjected to careful physicochemical analysis, which confirmed the preparation of nanocrystalline hydroxyapatite containing 0.26 wt% of Mg and 0.59 wt% of Si.

### 3.2. Preparation of the Composite Beads

Three different composite beads were prepared using previously synthesized Mg,Si-HA powder, sodium alginate (SA) (Sigma Aldrich, St. Louis, MO, USA) and two additives: chondroitin sulphate sodium salt (CS) (TCI, Tokyo Chemical Industry Co., Tokyo, Japan) and keratin from wool (KER) (TCI, Tokyo Chemical Industry Co., Tokyo, Japan). The drug used was raloxifene hydrochloride (RAL) (TCI, Tokyo Chemical Industry Co., Tokyo, Japan). The detailed composition of the granules is shown in Table 2.

Our aim was to produce materials that resemble natural biocomposite bone tissue [48,49,50,51]. This is why the mineral phase (Mg,Si-HA powder) was added to an organic matrix, composed of naturally occurring biopolymers. The preparation of the above beads involved the following steps: Mg,Si-HA and one of the selected additives (i) CS, (ii) KER, or (iii) CS/KER (a mixture of both in 1:1 weight ratio) were added to the aqueous sodium alginate solution. Raloxifene hydrochloride (RAL) powder was then added to the resulting suspension and mixed thoroughly. The suspension was added dropwise through a syringe into 6.4 wt% solution of Mg(NO_3_)_2_∙6H_2_O (Sigma Aldrich, St. Louis, MO, USA) dissolved in a mixture of distilled water and 96% ethanol (EMPROVE, Merck, Germany) in a 60:40 volume ratio. Beads were left for 15 min in the solution for cross-linking and to incorporate additional Mg^2+^ ions on the surface of the beads. The beads were named as follows: CS-RAL, KER-RAL, and CS/KER-RAL.

The obtained granules were then washed several times with distilled water and air dried at room temperature. After the cross-linking, the residual solution was extracted to examine the drug concentration in order to assess incorporation efficiency and raloxifene loss at the granule production stage. The raloxifene content in the cross-linking solution did not exceed 1 mg, so the raloxifene loss was below 1%. The dried beads were further subjected to physicochemical, mechanical, and biological analyses. In the same manner, control granules without raloxifene were obtained and named as follows: CS-c, KER-c, and CS/KER-c.

### 3.3. Scanning Electron Microscopy (SEM)

In order to analyze the morphology of the obtained samples, a SEM microscope JSM 6390 LV JEOL (JEOL, Tokyo, Japan) at 20 or 30 kV accelerating voltage was used. For SEM analysis, the surface of the beads was sputtered with gold in a vacuum chamber. The SEM images were taken from both the outer and the inner surface after the cross-section of the granule. 

### 3.4. Porosity and Mechanical Strength

The mercury intrusion porosimetry method was used for analysis of the specific surface area and the degree of porosity of the obtained beads. The experiments were carried out using an Autopore IV 9510 instrument (Micromeritics, Norcross, GA, USA), which enables measurements of mercury intrusion pressure in the range 0.0035 to 400 MPa. This method is based on applying controlled pressure to a sample which is immersed in mercury. Briefly, mercury, as a non-wetting liquid is forced into the pores by external pressure. The pressure required to intrude into the pores is inversely proportional to the size of the pores. The dried pieces of tested sample were placed in a measuring vessel (penetrometer) and degassed to a pressure of 50 mmHg. Volumes and pore size distributions were calculated using the Washburn equation. 

The beads were also tested to determine the mechanical crushing strength by determining the destructive force of the granules using the Tinius Olsen H10K-S instrument (Tinius Olsen, Horsham PA, USA). Each bead was placed between the stationary plate and the moving measuring head and subjected to a pressure test whilst moving the head at a speed of 5 mm/s. The mechanical strength is a ratio of the pressure at which the bead is destroyed (N) and the diameter of the bead (mm).

### 3.5. In Vitro Release of Mg and Si Ions from Mg,Si-HA Powder and Beads

In vitro release of Mg^2+^ and SiO_4_^4−^ ions from Mg,Si-HA powder and from the obtained beads was evaluated as follows: 1 g of the sample was filled with 12 mL of phosphate buffer saline (PBS) of pH 7.4 and put into a water bath at 37 °C under a gentle stirring. The measurements of the ions released from powder and beads were carried out for four and three weeks, respectively. Sample aliquots of 5 mL were taken at appropriate time intervals, and each sample was replaced with a portion of fresh buffer. For the quantitative measurement of magnesium and silicon released from the samples, the inductively coupled plasma optical emission spectrometer ICP-OES iCAP 7400 Duo Spectrometer (Thermo Scientific, Waltham, MA, USA) was used.

### 3.6. In Vitro Release of Raloxifene Hydrochloride from Beads

In vitro release of RAL from beads was evaluated in Falcon 50 mL tubes. The studies were performed in PBS of pH 7.4 and ethanol solution (1:1 weight ratio) to create the sink conditions since RAL is poorly soluble in water [52,53,54]. For the analysis, 0.3 g of each sample was immersed in 50 mL of release medium in the bath shaker and stirred at 100 rpm at 37 °C. Sample aliquots of 5 mL were withdrawn at regular time intervals. Each sample was kept in a separate tube to avoid multiple repeated medium changes. Since the RAL solutions are not stable, the experiment was planned to finish and take samples of all time intervals at the same time. Release was started with the longest measurement (12 weeks) and successive samples were inserted over time. At the end of the experiment, all the samples were filtered through a membrane syringe filter with pore size of 0.8 μm. The samples were then analyzed by the UV-Vis method using a Shimadzu UV-1800 spectrometer (Shimadzu, Kyoto, Japan). The absorbance was measured at 291 nm and all measurements were performed in triplicate (*n* = 3).

The percentage of drug released at each time point was calculated according to Equation (1):

Drug release (%) = drug in solution [µg/mL]/initial drug content in the sample [µg/mL] × 100%(1)

### 3.7. In Vitro Cytotoxicity Assessment

The in vitro cytotoxicity assessment was conducted using a normal human fetal osteoblast cell line (hFOB 1.19, ATCC-LGC Standards, Kiełpin, Poland) and a human osteosarcoma cell line (Saos-2, ATCC-LGC Standards, Kiełpin, Poland). The 1:1 mixture of DMEM/Ham’s F12 growth medium without phenol red (Sigma-Aldrich Chemicals, St. Louis, MO, USA) containing 100 µg/mL streptomycin, 100 U/mL penicillin, 300 µg/mL G418 (Sigma-Aldrich Chemicals, St. Louis, MO, USA), and 10% fetal bovine serum (PAN-Biotech GmbH, Aidenbach, Germany), was used as the culture medium for hFOB 1.19, whereas McCoy’s 5A medium (ATCC-LGC Standards, Kiełpin, Poland) containing 100 µg/mL streptomycin, 100 U/mL penicillin, and 15% fetal bovine serum was used as the culture medium for Saos-2. The hFOB 1.19 and Saos-2 cells were cultured in a humidified atmosphere of 5% CO_2_ at 34 °C and 37 °C, respectively. 

The cytotoxicity evaluation was conducted according to ISO 10993-5:2009 standard against hFOB 1.19 and Saos-2 cells. The MTT assay (Sigma-Aldrich Chemicals, St. Louis, MO, USA) was conducted using 24-h granule extracts as described earlier [55]. Then, 100 μL/well of cell suspension (2 × 10^5^ cells/mL and 3 × 10^5^ cells/mL of hFOB 1.19 and Saos-2, respectively) was seeded into 96-well polystyrene plates. After 24 h of culture, the culture medium was replaced with 100 µl of granule extracts. Polypropylene extract served as a negative control for cytotoxicity. The MTT test was performed as described earlier after 48-h exposure to the extracts [45]. Afterwards, the viability of hFOB 1.19 and Saos-2 cells was calculated according to Equation (2):

Cell viability (%) = sample OD/negative control OD × 100%(2)

## 4. Conclusions

The results enable us to conclude that the obtained granules are materials with favorable parameters, enabling the gradual, local delivery of medicinal substances to the immediate surroundings of the diseased tissue. Results revealed that the beads CS/KER-RAL, composed of both CS and KER, possessed the most sustained drug release and no burst release effect, compared to single-component samples (CS and KER). The obtained results may constitute a starting point for more extensive research, with particular emphasis on in vivo biological tests. Drug delivery systems releasing raloxifene directly to the bone tissue could be a beneficial alternative to its current route of administration. 

## Figures and Tables

**Table 1:** Porosity results of CS-RAL, KER-RAL, and CS/KER-RAL beads measured by the Hg intrusion technique.

Sample	S_Hg_ [m^2^/g]	ρ_b_ [g/cm^3^]	ρ_p_ [g/cm^3^]	P [%]	V_t_ [cm^3^/g]	V_me_ [cm^3^/g]	%V_me_ [%]	CR [N/bead]
	±0.1	±0.05	±0.05	±0.1	±0.001	±0.001	±0.1	±0.1
CS-RAL	47	1.5	1.9	19	0.12	0.08	63	139
KER-RAL	71	1.4	1.7	20	0.15	0.11	74	137
CS/KER-RAL	45	1.5	1.8	13	0.09	0.06	67	177

S_Hg_, surface area; ρ_b_, apparent density; ρ_p_, true density; P, porosity; V_t_, total pore volume; V_me_, mesopores volume; %V_me_, percentage of mesopores; CR, crush resistance.

**Table 2:** Bead composition.

Sample	Mg,Si-HA (g)	SA (g)	CS (g)	KER (g)	RAL (mg)
CS-RAL	2.8	2.0	1.2	-	100.0
KER-RAL	2.8	2.0	-	1.2	100.0
CS/KER-RAL	2.8	2.0	0.6	0.6	100.0
CS-c	2.8	2.0	1.2	-	-
KER-c	2.8	2.0	-	1.2	-
CS/KER-c	2.8	2.0	0.6	0.6	-

CS, chondroitin sulphate; KER, keratin; RAL, raloxifene hydrochloride; c, control.
